# Editorial: The Immunology of Adverse Drug Reactions

**DOI:** 10.3389/fimmu.2022.863414

**Published:** 2022-02-18

**Authors:** Patricia T. Illing, Nicole A. Mifsud, Michael R. Ardern-Jones, Jason Trubiano

**Affiliations:** ^1^ Infection and Immunity Program, Monash Biomedicine Discovery Institute and Department of Biochemistry and Molecular Biology, Monash University, Clayton, VIC, Australia; ^2^ Clinical Experimental Sciences, Faculty of Medicine, University of Southampton, Southampton, United Kingdom; ^3^ Department of Dermatology, University Hospitals Southampton NHS Foundation Trust, Southampton, United Kingdom; ^4^ Centre for Antibiotic Allergy and Research, Department of Infectious Diseases, Austin Health, Heidelberg, VIC, Australia

**Keywords:** adverse drug reaction, T cells, human leukocyte antigen, MRGPRX2, drug allergy, drug hypersensitivity

## Introduction

The immune system has evolved for both breadth and specificity of recognition to protect the body against a wide array of infectious and oncogenic challenges. Unfortunately, this recognition can also extend to certain therapeutic drugs causing drug hypersensitivity in affected individuals. These unwanted responses range in both severity and pathways of immune activation, eliciting deleterious, and in some cases potentially fatal, immune responses. Such adverse events place significant strain on health care systems and prevent use (in susceptible individuals) of key medications that are well tolerated by most patients at therapeutic doses. Here, we bring together experts in the field of adverse drug reactions, incorporating both clinical and laboratory-based researchers, addressing critical areas of prediction, diagnosis and mechanistic understanding of these reactions.

## T Cell-Mediated Drug Hypersensitivity

10 articles within this collection focus primarily on T cell-mediated drug hypersensitivity reactions (DHRs), also termed delayed-type drug hypersensitivity or type IV drug hypersensitivity (under the Gell and Coombs classification) (1). T cell-mediated DHRs involve Human Leukocyte Antigen (HLA)-dependent activation of T cells induced by the culprit drug and/or a metabolite *via* a range of mechanisms including: i) generation and presentation of covalently drug modified peptides (*i.e.* hapten/prohapten model) (2, 3), ii) labile interaction of the drug/metabolite with the HLA and/or TCR to trigger T cell activation (*i.e.* p.i. concept) (4) and iii) interaction of the drug within the HLA peptide-binding cleft to change the array and conformation of HLA-bound peptides (*i.e.* altered peptide repertoire) (5, 6). Furthermore, many of these reactions are associated with distinct HLA alleles, suggesting specific interactions and that HLA screening could be used as a predictive tool to prevent prescription to individuals carrying risk alleles. However, as discussed by Li et al., this is not an effective solution for many HLA-associated adverse reactions due to negative predictive values less than 100% and low positive predictive values, necessitating large numbers of individuals to be tested in order to prevent a single adverse event. In light of this, Li et al. explore the evidence for further risk modifiers within the HLA peptide presentation (Endoplasmic Reticulum aminopeptidase 1 [ERAP1] polymorphism) and recognition (T cell receptors [TCRs] and Killer-cell Immunoglobulin-like Receptors [KIRs]) pathways. Furthermore, they examine the concept of “dynamic risk” engendered by epigenetic changes, infection, and medications such as checkpoint inhibitors.

Original research articles by Pratoomwun et al., Puig et al., Mifsud et al., and Illing et al. focus on defining the molecular mechanisms of HLA-associated adverse reactions to co-trimoxazole, flucloxacillin, carbamazepine and abacavir, respectively. The study by Pratoomwun et al. explores T cell responses in two HLA-B*13:01^+^ co-trimoxazole (combination sulfamethoxazole [SMX] and trimethoprim) hypersensitive patients. Surprisingly, HLA-B*13:01-restricted CD8^+^ T cells were not identified, instead the metabolite (nitroso-SMX) and SMX responsive T cell clones were CD4^+^ and HLA class II restricted, suggesting HLA-B*13:01 was not directly involved in presentation of the immunogenic antigens. Aligned with previous studies (7, 8), there were multiple modes of presentation observed, consistent with covalent modification of presented peptides by nitroso-SMX (in either antigen processing dependent or independent manners) and recognition of soluble SMX. To precisely examine the presented peptides at the cell surface, Puig et al., Mifsud et al., and Illing et al. combine immunopeptidomics with functional analyses to define immune activation *via* the presentation of covalently drug-modified peptides (Puig et al.; HLA-B*57:01-mediated presentation of flucloxacillin modified peptides), labile drug interaction (Mifsud et al.; HLA-B*15:02 and carbamazepine) and an altered peptide repertoire (Illing et al.; HLA-B*57:01 and abacavir) to extend previous mechanistic studies of these reactions (5, 6, 9–11).

In line with the review by  Li et al., these studies each demonstrate the influence of antigen processing and presentation including the HLA-TCR/KIR axis. In addition to the identification of covalently modified peptides capable of eliciting T cell responses in transgenic HLA-B*57:01^+^ mice, Puig et al. speculated that broader changes in the immunopeptidome were due to altered peptide processing, and that the location of identified modifications could alter KIR recognition. Mifsud et al. explored highly focused, private TCR responses to carbamazepine in severe DHRs, and Illing et al. assessed the influence of tapasin and abacavir on peptide loading onto (and dissociation from) HLA-B*57:01, highlighting the potential for antigen processing and presentation machinery to shape the drug-induced immunopeptidome.

Moving beyond the mechanisms for T cell activation, reviews by Schunkert et al. and Bechara et al. focus on the nature of the T cells responding to drug/chemical stimulation. Bechara et al. discuss the role of the naïve T cell repertoire in the recognition of drugs and chemicals and the persisting unknowns of the drug-responsive naïve T cell repertoire such as frequency, public vs private TCRs and origin during thymic selection. Schunkert et al. review the evidence for a role for skin resident memory T cells (T_RM_) in delayed type hypersensitivity reactions, speculating on the mechanisms by which drug-responsive T_RM_ may be generated and become lodged in the skin. They highlight important challenges in phenotypic analysis which hinder understanding of T_RM_ in delayed type hypersensitivity and posit that novel mouse models of delayed type hypersensitivity [*e.g.* (12)] provide a road forward for dissecting the contribution of T_RM_ by enabling exploration of rechallenge.

DHRs often clinically present as complex immunopathologies. To address this, Thompson et al., Hammond et al., and Hertzman et al. focus on tools and workflows for accurate diagnosis, prediction and multi-omic analysis of DHRs. Through a series of case studies, Thompson et al.  present a diagnostic workflow (incorporating patch and intradermal testing) for the assessment of β-lactam cross-reactivity or co-reactivity in non-Stevens-Johnson Syndrome/toxic epidermal necrolysis (SJS/TEN) severe cutaneous adverse reactions to determine whether broad β-lactam avoidance is necessary or careful testing of alternative β-lactams may be tolerated. Hammond et al. explore the utility of different *in vitro* diagnostic assays for the identification of culprit drugs in T cell-mediated drug hypersensitivity as well as preclinical assays to predict immunogenicity. Finally, Hertzman et al. present a tool for the analysis and visualisation of single cell TCR-seq, RNA-seq and Cellular Indexing of Transcriptomes and Epitopes by Sequencing (CITE)-seq data to enable researchers to take advantage of the exciting capacities of these technologies to reveal signatures of distinct cell subsets involved in the pathogenesis and prevention of different adverse reactions.

## Mas-Related G Protein-Coupled Receptor X2

Two review articles (Mackay et al. and McNeil) focus on the emerging role for Mas-related G protein-coupled receptor X2 (MRGPRX2) mediated mast cell activation in antibody-independent immediate hypersensitivity reactions. With a focus on anaphylaxis, Mackay et al. discuss strategies to pinpoint the role of MRGPRX2 and isolate biomarkers, further considering roles for MRGPRX2 agonists and antagonists in therapeutic applications. Moreover, McNeil interrogates the relationship between peak serum concentrations, as well as localised areas of increased concentration in specific tissues/locations, and the EC_50_ of known MRGPRX2 agonists in mild-moderate immediate hypersensitivity reactions. Both studies highlight the need for further investigation to understand the role of MRGPRX2 in adverse events and to provide clear diagnostic criteria.

## Concluding Remarks

Collectively these articles highlight that, as for many fields, phenotypic, diagnostic, predictive and mechanistic studies traversing the clinic to the laboratory bench (and computer) and back are critical to understanding the complex biological interactions that characterise drug hypersensitivity. Future genetic and mechanistic analyses will build upon clinical observations, with the capacity to identify new biomarkers and signatures of disease that feedback to improve diagnosis, prediction, and prevention.

## Author Contributions

PTI drafted the manuscript. All authors edited and approved the manuscript.

## Conflict of Interest

PTI and NAM were authors of two papers within the collection (Illing et al. and Mifsud et al.).

The remaining authors declare that the research was conducted in the absence of any commercial or financial relationships that could be construed as a potential conflict of interest.

## Publisher’s Note

All claims expressed in this article are solely those of the authors and do not necessarily represent those of their affiliated organizations, or those of the publisher, the editors and the reviewers. Any product that may be evaluated in this article, or claim that may be made by its manufacturer, is not guaranteed or endorsed by the publisher.
